# Case Report: Tackling Complement Hyperactivation With Eculizumab in Atypical Hemolytic Uremic Syndrome Triggered by COVID-19

**DOI:** 10.3389/fphar.2022.842473

**Published:** 2022-02-28

**Authors:** Valentina Fanny Leone, Amantia Imeraj, Sara Gastoldi, Caterina Mele, Lucia Liguori, Carmelita Condemi, Piero Ruggenenti, Giuseppe Remuzzi, Camillo Carrara

**Affiliations:** ^1^ Unit of Nephrology, Azienda Socio-Sanitaria Territoriale Papa Giovanni XXIII, Bergamo, Italy; ^2^ Istituto di Ricerche Farmacologiche Mario Negri IRCCS, Milano, Italy

**Keywords:** eculizumab, coronavirus disease 2019 (COVID-19), severe acute respiratory syndrome coronavirus-2 (SARS-Cov-2), thrombotic microangiopathy, hemolytic uremic syndrome

## Abstract

Hemolytic uremic syndrome (HUS) is a rare life-threatening disease of unrestrained complement system dysregulation, microangiopathic hemolytic anemia, thrombocytopenia, and acute renal failure in genetically predisposed individuals. In this report, we describe two cases of SARS-CoV-2–associated HUS treated with eculizumab, a C5-blocking monoclonal antibody reported to be remarkably effective in the treatment of HUS. Detailed biochemical and genetic complement system analysis is reported, and the prompt clinical response after C5 pharmacological blockade is documented. Our report provides the rationale and supports the use of terminal complement pathway inhibition for the treatment of SARS-CoV-2–associated HUS.

## Introduction

Over 270 million people have been infected globally with severe acute respiratory syndrome coronavirus-2 (SARS-CoV-2), and over 5 million have died from coronavirus disease (COVID-19) since the first cases were detected in the city of Wuhan, China (https://coronavirus.jhu.edu/map.html, 2021)*.* SARS-CoV-2 invades the alveolar cells in the lungs, which is the virus’ portal of entry, and then it spreads to the rest of the body, causing, in most severely ill patients, multiorgan involvement and damage (Guan et al., 2020). Accumulating evidence indicates that the mechanisms of COVID-19–associated tissue injury are not primarily mediated by viral infection but rather are the result of the host immune response, which drives hypercytokinemia and inflammation (Chen et al., 2020)*.*

Unrestrained complement system activation is emerging as a key factor of endothelial dysfunction, thrombosis, and organ damage in patients with COVID-19 (Afzali et al., 2021; Aiello et al., 2021)**.**

In particular, increased levels of C5a and strong deposits of C5b-9 have been detected respectively in the serum, lung parenchyma, and vessels of COVID-19 patients, highlighting the role of the complement terminal pathway in the critical phase of the viral infection (Diao et al., 2021) (Carvelli et al., 2020). Higher baseline plasma concentrations of C3a and sC5b-9 were observed in COVID-19 patients experiencing a thromboembolic event, and sC5b-9 concentrations correlated with D-dimer levels (de Nooijer et al., 2021)**.** Furthermore, fibrin and intense C5b–9 immunostaining were found in the kidney glomeruli and cardiac microthrombi (Pfister et al., 2020; Pellegrini et al., 2021)**.**

The C5 complement-blocker eculizumab proved to be an effective weapon in complement-related disorders and radically improved outcomes of patients with atypical hemolytic uremic syndrome (HUS) (Gruppo and Rother, 2009; Legendre et al., 2013; Noris et al., 2012)*.* These evidences provided the rationale for studies evaluating the potential therapeutic effect of complement inhibition by the C5 blocker eculizumab in patients with COVID-19 (Annane et al., 2020). It is to be noted that a recent cohort study in patients with severe COVID-19 showed that complement inhibition was associated with prompt improvement in respiratory dysfunction and decreased the combined endpoint of long-term mortality and chronic complications as compared to contemporary similar controls (Ruggenenti et al., 2021). However, other studies failed to demonstrate a clinical benefit of C5 inhibition (https://ir.alexion.com/news14releases/news-release-details/alexion-provides-update-phase-3-study-ultomirisr-ravulizumab). Conceivably, eculizumab should prove to be even more effective in COVID-19 patients with genetically determined abnormalities in the complement system (as in atypical HUS) and those who present with clinical features of thrombotic microangiopathy (TMA) characterized by the triad of thrombocytopenia, microangiopathic hemolytic anemia, and evidence of organ injury. HUS has been exceptionally reported in association with COVID-19 (Jhaveri et al., 2020; Mat et al., 2021; Safak et al., 2021; Ville et al., 2021; Boudhabhay et al., 2021). It could be hypothesized that SARS-CoV-2 may act as a trigger of complement activation in genetically predisposed individuals, as occurs in complement-associated HUS and occasional cases of Shiga toxin–producing *Escherichia coli–*associated HUS (STEC-HUS) (Alberti et al., 2013)**.**

Here, we describe two additional patients infected with SARS-CoV-2 who developed HUS. Biochemical and genetic complement system studies and the rapid clinical response after C5 pharmacological blockade support the use of eculizumab in this setting.

## Case Presentation

### Patient 1

A 77-year-old female with a history of arterial hypertension, rheumatoid arthritis, and chronic hepatitis B presented to our emergency department after a 10-day history of asthenia, vomiting, and progressive oliguria. At presentation, she was afebrile, and arterial blood pressure was in good control. Clinical examination of the lungs was relevant for diffuse bilateral crackles, while the rest of general examination was unremarkable. She was then admitted to the Infectious Disease Unit.

Chest X-ray showed interstitial bilateral opacities, suggesting SARS-CoV-2 interstitial pneumonia. The diagnosis of SARS-CoV-2 infection was confirmed by a nasopharyngeal swab on real-time reverse transcription polymerase chain reaction (RT-PCR). Laboratory tests at presentation revealed acute renal failure, while blood count was normal and C-reactive protein (CPR) only mildly elevated. From day 3, the patient developed severe anemia and thrombocytopenia with positive markers of intravascular hemolysis, such as increase of lactate dehydrogenase and haptoglobin consumption, with a few schistocytes that were evidenced in the blood smear (Table 1). D-dimer was markedly elevated, while prothrombin (PT) and partial thromboplastin time (PTT) were normal and Coombs test negative. ADAMTS13 activity was normal with no ADAMTS13 inhibitor autoantibodies identified. Heparin-induced antibodies tested negative. There was no C3 or C4 hypocomplementemia. The genetic assessment of complement regulatory genes and ADAMTS13 through next-generation sequencing (NGS) was negative, and no risk haplotypes were found, although an heterozygous variant of unknown significance (VUS) was identified in the CFHR4 gene (the synonymous variant c.1234C > A, p.Arg412Arg, maxAF 0.0031 in gnADgen_OTH), which is predicted (by Human Splicing Finder) to potentially alter splicing by activating a cryptic splice acceptor site. Additional genetic analyses, conducted through multiplex ligation–dependent probe amplification (MLPA), revealed the polymorphic heterozygous deletion of CFHR3/CFHR1 genes.

**TABLE 1:** Laboratory values of the two patients at baseline.

Variable (*pre eculizumab*)	Patient 1	Patient 2
White cell count (per μl)	12,460	6,790
Red cell count (per μl)	2,620	2,960
Hemoglobin (g/dl)	7.8	9
Hematocrit (%)	21.3	27.2
Platelet count (per μl)	56,000	99,000
Creatinine (mg/dl)	9.43	5.88
Urea nitrogen (mg/dl)	204	212
Sodium (mmol/L)	147	150
Potassium (mmol/L)	3.5	4.2
Total bilirubin (mg/dl)	0.7	1
Aspartate aminotransferase (U/liter)	8	47
Alanine aminotransferase (U/liter)	13	16
D-dimer	4,234	2058
Prothrombin time (PT) (ratio)	1.13	1.88*
Activated partial thromboplastin time (ratio)	1.18	1.55
Lactate dehydrogenase (U/liter)	548	1758
Haptoglobin	0.04	<0.01
Schistocytes	Rare	Rare
C-reactive protein (mg/dl)	9.4	11.5
C3 (mg/dl)	81	72
C4 (mg/dl)	21	19
Ab anti-ADAMTS13 (U/ml)	7	7
ADAMTS 13 inhibitory activity	18%	27%
Coombs’ test	Negative	Negative
Antinuclear antibodies (ANAs)	Negative	Negative
Anti-DNA	Negative	NA
Extractable nuclear antigen antibodies (ENAs)	Negative	NA

**FIGURE 1:**
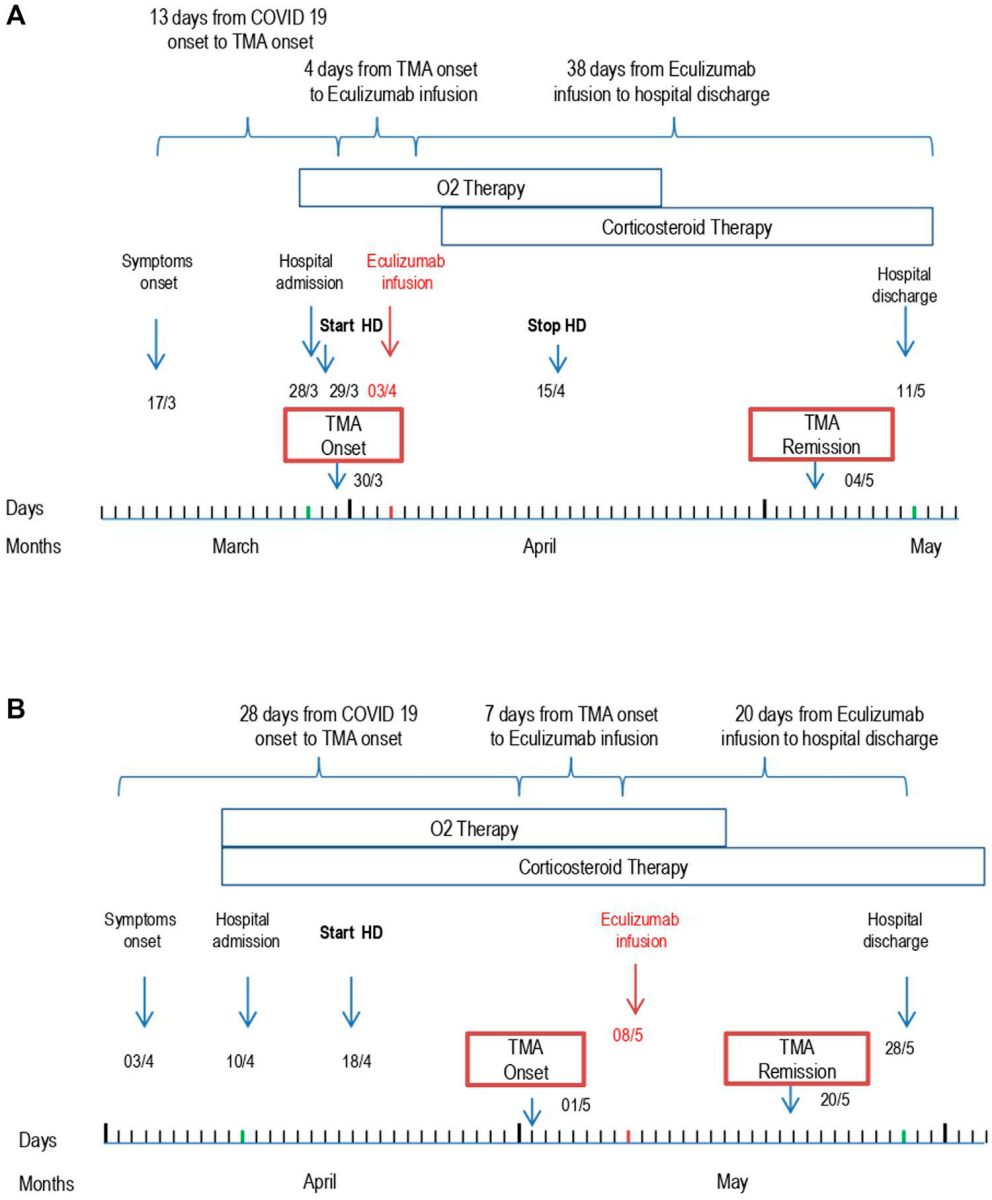
Patient 1 **(A)** and Patient 2 **(B)** outcome from symptom onset to hospital discharge. Abbreviations: Thrombotic microangiopathy (TMA); Hemodialysis (HD).

Due to worsening respiratory failure, the patient was treated with oxygen therapy through nasal cannulas and a course of pulse and then oral steroids and large spectrum antibiotic therapy was initiated. Anticoagulant therapy with heparin, which was rapidly initiated at the time of patient admission, was discontinued on day 3 due to worsening thrombocytopenia. Supportive care management of acute renal failure being ineffective, renal replacement therapy was started on day 2. On day 8, after admission, the patient was treated with eculizumab (900 mg) (Figure 1A).

The blood tests showed prompt improvement of platelet count and normalization of hemolysis parameters. Hemodialysis was stopped on day 20, given the amelioration of kidney function and urine output. The respiratory insufficiency gradually resolved. The patient was discharged on day 39 in good clinical conditions but with mild residual renal dysfunction (serum creatinine 2.47 mg/dL). Plasma levels of the terminal complement complex sC5b-9 measured 3 months after onset were normal (157 ng/ml, n.v. <300 ng/ml).

### Patient 2

A 79-year-old female with a past history of valvular heart disease, initial cerebral vasculopathy, and stage IV chronic kidney disease (CKD) presented to our emergency department with low-grade fever, shortness of breath, oliguria, and gross hematuria. Arterial blood pressure was only mildly elevated. The pulse oximeter demonstrated moderate hypoxemia. She was then admitted to the Infectious Disease Unit.

Initial laboratory tests showed severe anemia, elevated CRP, and modest elevation of D-dimer. Infection by SARS-CoV-2 was confirmed by RT-PCR performed on nasal swab. Chest X-ray revealed bilateral accentuation of the pulmonary interstitium. Diffuse “ground glass” opacities were confirmed at a subsequent chest CT-scan. A renal ultrasonogram showed reduced kidney size with thinned cortical thickness. On day 11, a cerebral CT scan was performed due to a confusional state and an absence episode with slight deviation of the left buccal rim. However, no recent parenchymal density alterations were found.

**FIGURE 2:**
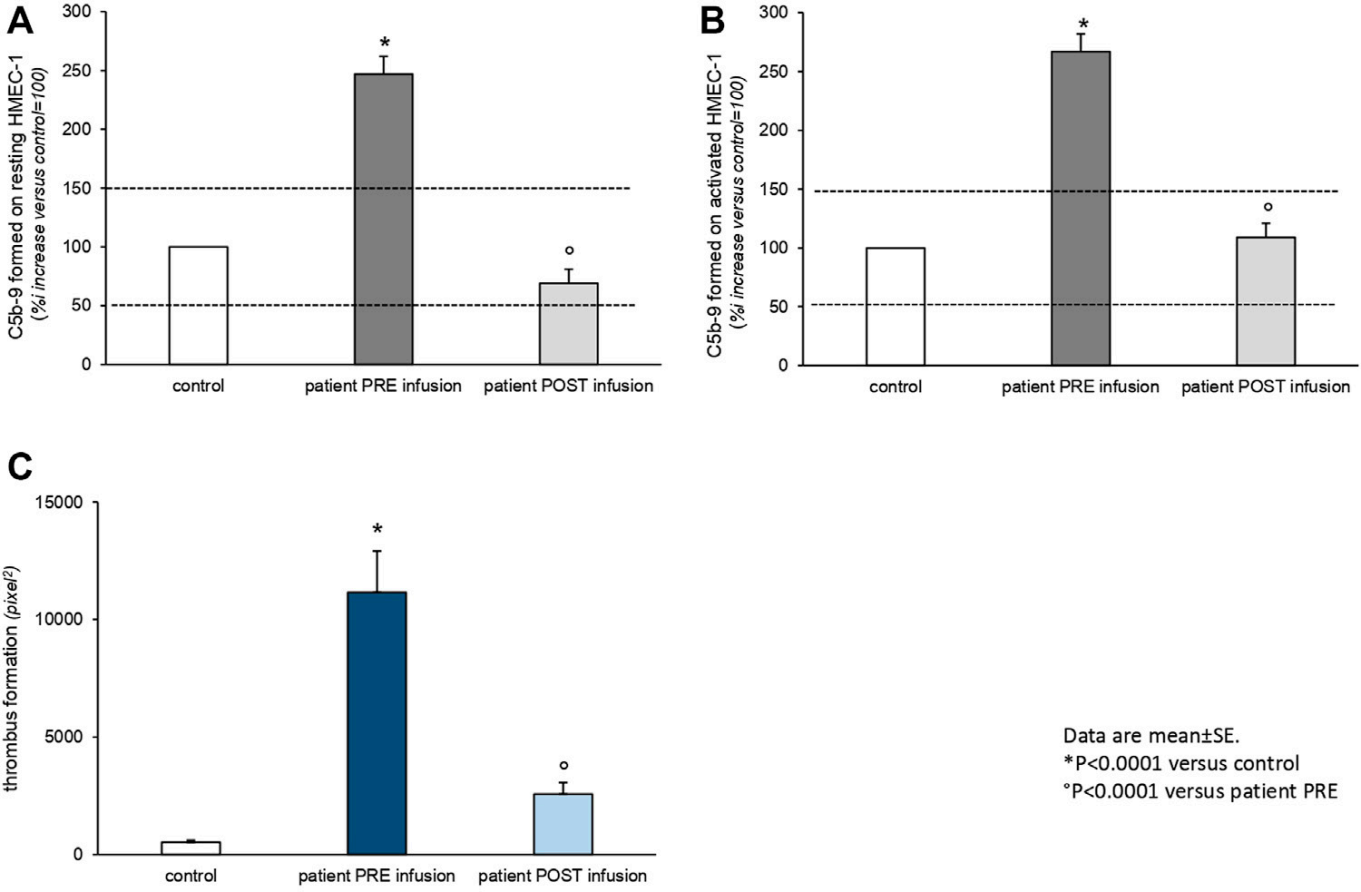
*Ex vivo* C5b-9 deposition **(A,B)** and thrombus formation **(C)** on the cultured human microvascular endothelial cell (HMEC-1) line induced by serum of Patient 2 collected before (PRE infusion) and 10 days after (POST infusion) eculizumab treatment. **(A,B)**. HMEC-1 resting **(A)** or activated with ADP **(B)** were incubated for 2 h with serum (diluted 1:2 with test medium, HBSS with 0.5% BSA) from the patient or with a control serum pool. At the end of incubation, the cells were washed, fixed, and stained with rabbit anti-human complement C5b-9 complex antibody followed by the FITC-conjugated secondary antibody. Fluorescence microscopy was used to view the fluorescent staining on the endothelial cell surface, and the HMEC-1 area covered by C5b-9 staining was calculated by automatic edge detection (ImageJ software) in 15 high-power fields. For each sample, the highest and lowest values were discarded, and the mean of the other 13 fields was calculated and values were expressed as the percentage of C5b-9 deposits induced by a pool of sera from 10 healthy controls run in parallel (reference 100%). Dashed lines indicate the upper and lower limit of normal range. **(C)** HMEC-1 were activated with ADP and exposed for 2 h to serum (diluted 1:2 with test medium, HBSS with 0.5% BSA) from the patient or with a control serum pool. Perfusion of heparinized whole blood (heparin 10 U/ml) from a healthy subject (added with the fluorescent dye mepacrine 10 µM, to label platelets) was then performed in a thermostatic flow chamber (37°C) in which one surface of the perfusion channel was a glass slide seeded with a monolayer of endothelial cells at a constant flow rate of 1,500 s^−1^ (60 dynes/cm^2^). After 3-min perfusion was stopped, the slide with the endothelial cell monolayer was dehydrated and fixed in acetone for 20 min. The slides were examined under a confocal inverted laser microscope. Fifteen fields for each slide were systematically digitized along the surface, and the area covered by thrombi was quantified by ImageJ (NIH, Bethesda, MD) and expressed as pixel^2^ per field analyzed. For each sample, the mean of 15 fields (excluding the lowest and the highest values) was calculated. Data are reported ±SE. *p < 0.0001 versus control serum pool; *p* < 0.0001 *versus* patient PRE infusion. Statistical analysis: ANOVA.

From day 21, laboratory tests demonstrated signs of microangiopathic hemolysis with down-trending platelets and hemoglobin, elevation of lactate dehydrogenase, undetectable haptoglobin levels, and presence of schistocytes on blood smear (Table 1)**.** ADAMTS13 activity was normal, with no ADAMTS13 inhibitor autoantibodies identified. There was moderate C3 hypocomplementemia with C4 being in the normal range. The plasma levels of sC5b-9 were very elevated (1,190 ng/ml). Marked C5b-9 deposition was found on the microvascular endothelial cells (247% on resting and 267% on activated endothelial cells, normal values < 150%) (Noris et al., 2014) (Figure 2)**.** The genetic assessment of complement regulatory genes through NGS revealed a heterozygous rare missense variant in the gene encoding C3 (c.26T > C that causes the p. Leu9Pro, max AF 0.001 in ExAC_NFE), that is predicted to be likely pathogenetic *in silico* (CADD 20), as well as the heterozygous H3 risk haplotype in the gene encoding CFH. MLPA analyses on CFH/CFHR genes did not reveal any abnormality.

The patient was treated with intravenous steroids, furosemide, and empiric antibiotic therapy. Oxygen therapy was initiated through nasal cannulas. Anticoagulant therapy with heparin was administered. Due to progressive worsening of renal function with anuria, renal replacement therapy was initiated on day 8. A single dose of 900 mg eculizumab was given on day 27 shortly after the appearance of signs of TMA (Figure 1B). After eculizumab administration, laboratory work-up showed progressive elevation of platelets, hemoglobin levels, and disappearance of schistocytes from the blood smear. Detailed complement testing was again performed 10 days after eculizumab infusion and evidenced the normalization of C5b-9 deposition on the microvascular endothelial cells (Figure 2). The control chest X-ray performed on day 32 showed initial (but not complete) improvement of the lung interstitium. Control nasopharyngeal swabs were performed on days 28 and 30 with negative results. Unfortunately, renal function failed to recover, and the patient was discharged (day 45) still on renal replacement therapy.

## Discussion

Here, we present the cases of two patients infected by SARS-CoV-2, who developed severe hemolytic anemia and worsening renal failure in the context of HUS. The patients were both treated with a single dose of eculizumab without concomitant therapeutic plasma exchanges. The use of the C5-inhibitor monoclonal antibody resulted in the progressive improvement of hematological parameters in both patients. On the other hand, while renal replacement therapy could be stopped in Patient 1 due to improving kidney function, Patient 2, who had previous evidence of stage IV CKD, remained dialysis-dependent. Due to the critical conditions and rapidly evolving clinical scenario, Patient 1 was treated empirically with eculizumab as soon as the diagnosis of HUS was established. Conversely, inspired by the favorable course of the first case, we set up a protocol aimed at understanding the potential mechanisms of SARS-CoV-2–associated HUS and therapeutic effect of C5 pharmacological blockade, which we have successfully applied to Patient 2 (Figure 2)**.** During the active phase of the disease, we were able to demonstrate higher than normal C5b-9 and thrombi deposition on the microvascular endothelial cells in Patient 2, which completely normalized after treatment with eculizumab. Genetic analysis revealed a heterozygous variant of unknown significance in the CFHR4 gene and polymorphic heterozygous deletion of CFHR3/CFHR1 genes in Patient 1 and detected a rare variant in the gene encoding for C3, considered likely pathogenic, as well as the H3 risk haplotype in the gene encoding for CFH in Patient 2. Thus, in both cases, we found genetic abnormalities in the complement system that could predispose to the development of HUS upon exposure to potential triggers such as certain infectious agents, drugs, or pregnancy (Noris et al., 2012)*.*

HUS represents a clinical manifestation of TMA. It is a rare life-threatening syndrome which results from platelet adhesion to the vascular endothelium and their aggregation and activation with formation of platelet thrombi in the microvasculature, leading to consumptive thrombocytopenia, microangiopathic hemolytic anemia, and acute renal failure (Remuzzi, 1987). STEC-HUS, also called typical HUS, is the most frequent form of infection-associated HUS (Johannes and Römer, 2010) ***.*** The toxin produced by the bacteria triggers endothelial complement deposition through the upregulation of P-selectin and possibly interferes with the activity of complement regulatory molecules. In addition, complement activation has been evidenced in children with STEC-HUS, in terms of higher plasma levels of the alternative complement activation products, such as C3b, C3c, C3d, and increased levels of C5 convertase and of the terminal complement complex sC5b-9 (Thurman et al., 2009; Ferraris et al., 2015; Westra et al., 2017)**.**

In atypical HUS, instead, complement activation occurs as a consequence of genetic dysregulation of the complement alternative pathway (Noris and Remuzzi, 2009)**.** In most cases, however, a trigger is required to develop disease manifestations. Environmental hits are likely to induce endothelial perturbation and complement activation, which in healthy individuals are self-limiting as a result of multiple, redundant, regulatory mechanisms (Noris et al., 2012)*.* An individual with genetic abnormalities affecting complement regulation is particularly vulnerable to complement attack. Once the complement cascade is activated beyond a critical threshold, C3b formation and deposition occur on the vascular endothelium, which leads to further complement activation through the alternative pathway self-amplifying loop, culminating in microangiopathic injury and thrombosis. Reports from large cohorts show that atypical HUS onset or relapse are often triggered by bacterial and viral infections (Fang et al., 2008; Noris et al., 2010)**,** as well as the occurrence of HUS in COVID-19 patients (El Sissy et al., 2021; Mat et al., 2021), and present data corroborate the hypothesis that SARS-CoV-2 triggers the development of renal TMA through activation of the complement alternative pathway.

The clinical manifestation of SARS-CoV-2–associated HUS, which is an extremely rare complication of SARS-CoV-2 infection (Jhaveri et al., 2020; Mat et al., 2021; Safak et al., 2021; Ville et al., 2021; Boudhabhay et al., 2021), should not be confounded with the much more common systemic coagulopathy that is frequently observed in patients with COVID-19 (Zhou et al., 2020; Tang et al., 2020; Wu et al., 2020). The coagulopathy related to COVID-19 may resemble several systemic coagulation disorders seen in other severe infections, such as disseminated intravascular coagulation (Gando et al., 2016; Levi and Thachil, 2020)*.* Nevertheless, the clinical presentation and laboratory features, at a closer look, may be distinctly different. The prothrombotic state in COVID-19 coagulopathy is associated with high incidence of venous thromboembolism with few hemorrhagic complications in the absence of a real consumptive coagulopathy (Levi and Thachil, 2020)*.* Changes in hemostatic parameters, represented by increase in D-dimer and fibrin/fibrinogen degradation products, indicate that the essence of SARS-CoV-2–related coagulopathy is massive fibrin formation. In comparison with bacterial sepsis–associated disseminated intravascular coagulation, prolongation of PT time is less frequent, and thrombocytopenia is uncommon in COVID-19 (Iba et al., 2020)*.* In addition, histopathology from postmortem samples of COVID-19 patients typically shows microvascular platelet-rich thrombi limited to the small vessels of the lungs (Fox et al., 2020)*.* In summary, SARS-CoV-2 coagulopathy appears to be largely dependent on the severity of endothelial damage triggered by the viral infection and may potentially affect any patient with severe COVID-19. Conversely, SARS-CoV-2–associated HUS is an extremely less frequent complication of SARS-CoV-2 infection that requires a genetic predisposition to result into clinically relevant endothelial damage and TMA (Noris et al., 2020)*.*

The evidence that complement blockade effectively controls microangiopathy and thrombus formation in atypical HUS, together with the demonstration of complement activation during STEC-HUS, has led to the first attempts of eculizumab use in STEC-HUS, although no systematic assessment of its efficacy in this specific form of HUS is yet available (Walsh and Johnson, 2019)*.* The good outcome of severe STEC-HUS cases treated with eculizumab provided the rationale for its use in COVID-19–associated HUS (Jhaveri et al., 2020; Mat et al., 2021; Safak et al., 2021; Ville et al., 2021; Boudhabhay et al., 2021).

The main limitation of our study is the lack of the *ex vivo* test of C5b-9 and thrombi deposition on microvascular endothelial cells for Patient 1. Efficacy of eculizumab in COVID-19–associated HUS needs to be confirmed in larger series of patients.

In conclusion, our case report emphasizes the importance of monitoring parameters of hemolysis in patients with COVID-19 to act promptly in the event that HUS develops during the disease. Once HUS is evidenced, we encourage considering complete genetic and biochemical complement system assessment, if possible. Eculizumab, however, should be started as soon as the clinical diagnosis of SARS-CoV-2–associated HUS is established, before target organs are irreversibly damaged by the microangiopathic process. Treatment with the C5-blocker, already validated in HUS and being studied in COVID-19, may play a pivotal role in those rare cases of COVID-19–associated HUS.

## Data Availability

The original contributions presented in the study are included in the article/Supplementary Material; further inquiries can be directed to the corresponding author.
